# Supplementary material for: Long-Term Vemurafenib Exposure Induced Alterations of Cell Phenotypes in Melanoma: Increased Cell Migration and Its Association with EGFR Expression
Source: Int J Mol Sci. 2019 Sep 11;20(18):4484. doi: 10.3390/ijms20184484 (PMC6770060; doi:10.3390/ijms20184484)
Supplement: Supplementary file 1 [file ijms-20-04484-s001.zip › Table S1.docx]

| **PRIMER** | **SEQUENCE** |
| --- | --- |
| SNAIL FOR | TAT GCT GCC TTC CCA GGC TTG |
| SNAIL REV | ATG TGC ATC TTG AGG GCA CCC |
| ZEB-1 FOR | CCA GTG GTC ATG ATG AAA ATG GAA CAC C |
| ZEB-1 REV | CAG ACT GCG TCA CAT GTC TTT GAT CTC |
| VIMENTIN FOR | GGC TCA GAT TCA GGA ACA GC |
| VIMENTIN REV | CTG AAT CTC ATC CTG CAG GC |
| N-CADHERIN FOR | GCA TCA TCA TCC TGC TTA TCC |
| N-CADHERIN REV | TTC TCC TCC ACC TTC TTC ATC |
| E-CADHERIN FOR | CAG AGC CTC TGG ATA GAG AAC GCA |
| E-CADHERIN REV | GGC ATT GTA GGT GTT CAC ATC ATC GTC |
| MMP1 FOR | TAC ATG CGC ACA AAT CCC |
| MMP1 REV | ACA GCC CAG TAC TTA TTC CC |
| MMP3 FOR | TGG GCC AGG GAT TAA TGG AG |
| MMP3 REV | GGG AGT GGC CAA TTT CAT GAG |
| FRA-1 FOR | ACA CCC TCC CTA ACT CCT TTC |
| FRA-1 REV | TGC TGC TAC TCT TGC GAT G |
| MITF FOR | AGA CAT GCG CTG GAA CAA GG |
| MITF REV | GAG CAA CAA ATG CCG GTT GG |
| EGFR TAQMAN | HS01076078_M1 |
| GAPDH FOR | AGC TCA CTG GCA TGG CCT TC |
| GAPDH REV | ACG CCT GCT TCA CCA CCT TC |
| β-ACTIN FOR | ACT CTT CCA GCC TTC CTT C |
| β-ACTIN REV | GAT GTC CAC GTC ACA CTT C |
