# Supplementary figures and images for: Long-Term Vemurafenib Exposure Induced Alterations of Cell Phenotypes in Melanoma: Increased Cell Migration and Its Association with EGFR Expression
Source: Int J Mol Sci. 2019 Sep 11;20(18):4484. doi: 10.3390/ijms20184484 (PMC6770060; doi:10.3390/ijms20184484)

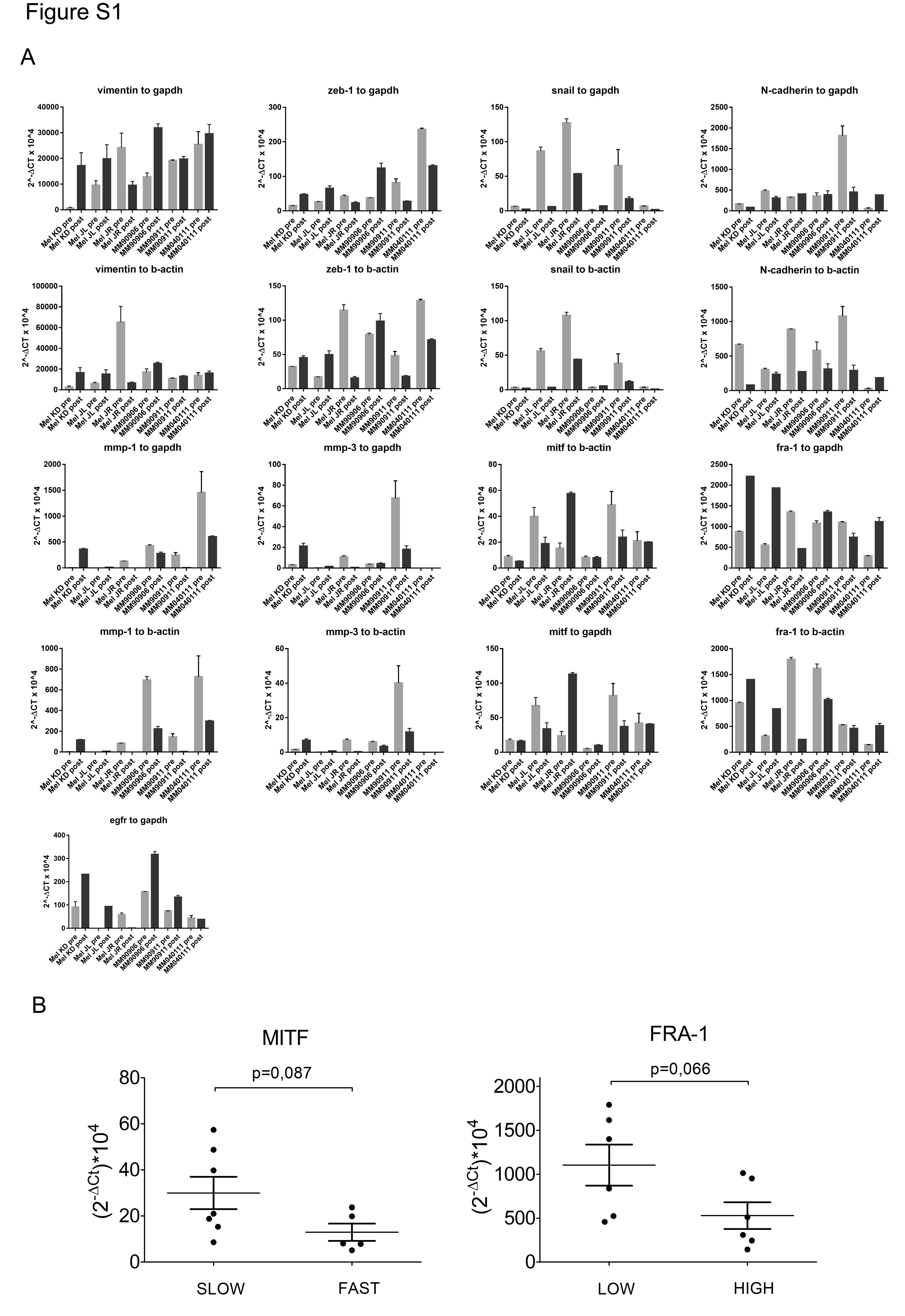

Supplement: Supplementary file 1 [file ijms-20-04484-s001.zip › Figure S1.jpg]

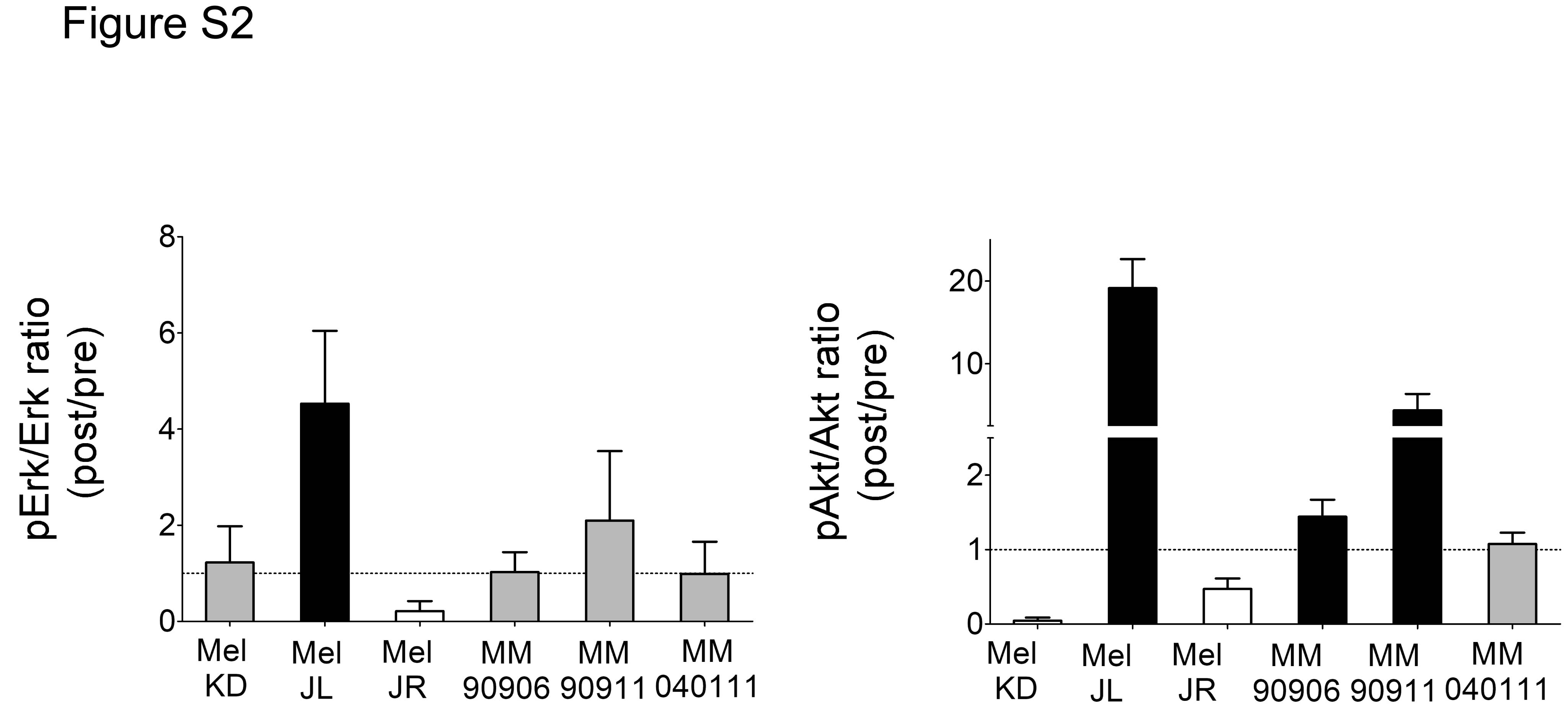

Supplement: Supplementary file 1 [file ijms-20-04484-s001.zip › Figure S2.jpg]

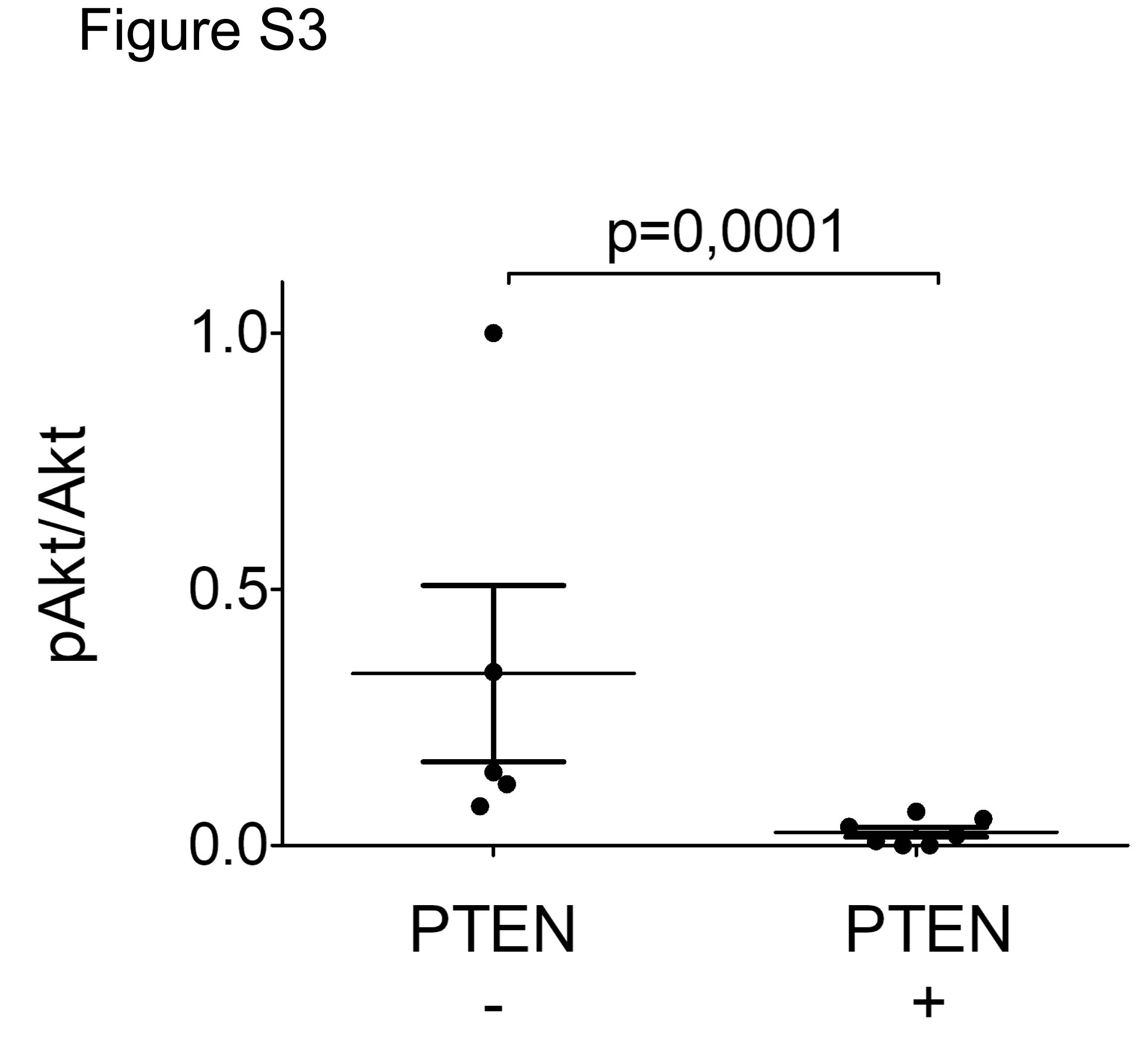

Supplement: Supplementary file 1 [file ijms-20-04484-s001.zip › Figure S3.jpg]

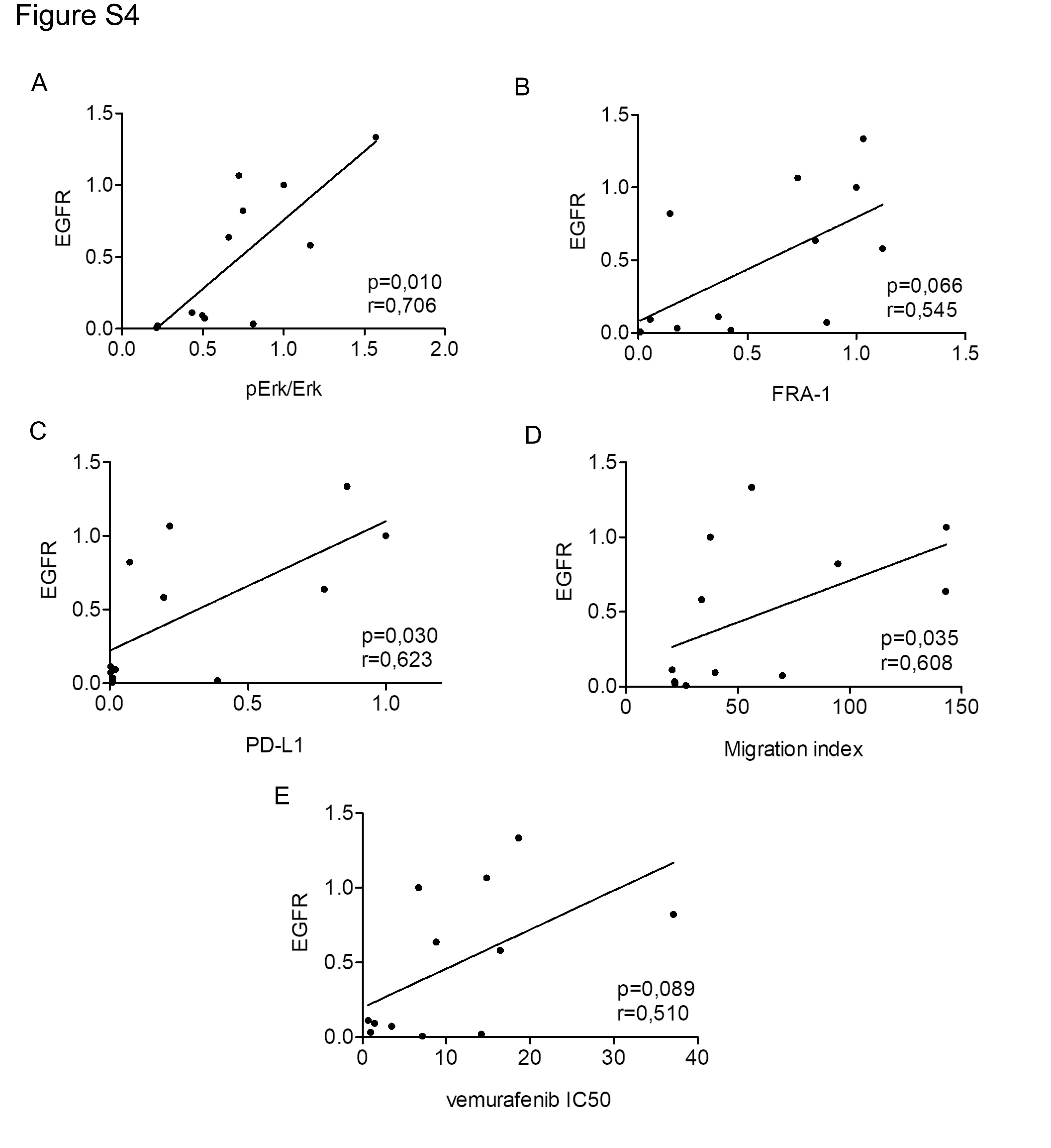

Supplement: Supplementary file 1 [file ijms-20-04484-s001.zip › Figure S4.jpg]
